# Healthcare professionals’ perceptions of a web-based application for using the new National Medication List in Sweden

**DOI:** 10.1177/20552076231171966

**Published:** 2023-05-08

**Authors:** Frida Bergman, Tora Hammar

**Affiliations:** 1 Department of Medicine and Optometry, 4180 Linnaeus University, Kalmar, Sweden; 2 eHealth Institute, Department of Medicine and Optometry, 4180 Linnaeus University, Kalmar, Sweden

**Keywords:** Shared medication list, web-based application, healthcare professionals, Sweden, user perspective, sociotechnical perspective, implementation, medication safety

## Abstract

**Objective:**

During the first stage of implementing the National Medication List in Sweden, a web-based application called Förskrivningskollen (FK) was launched. FK includes information about a patient's prescribed and dispensed medications, and it works as a backup system until the healthcare electronic health record (EHR) systems are fully integrated. The aim of this study was to examine the healthcare professionals’ experiences and perceptions of FK.

**Methods:**

The study applied a mixed methods approach, with statistics about the use of FK and a survey with open and closed questions. The respondents (n  =  288) were healthcare professionals who were users or potential users of FK.

**Results:**

Overall there was little knowledge about FK and uncertainty regarding working routines and the regulations connected to the application. Lack of interoperability with the EHRs made FK time-consuming to use. Respondents said that the information in FK was not updated, and they were concerned that using FK could lead to a false sense of security about the accuracy of the list. Most clinical pharmacists thought FK added benefit to their clinical work, while as a group, physicians were more ambivalent about FK's benefit.

**Conclusions:**

The concerns of healthcare professionals give important insights for future implementation of shared medication lists. Working routines and regulations linked to FK need to be clarified. In Sweden, the potential value of a national shared medication list will probably not be realized until it is fully integrated into the EHR in a way that supports healthcare professionals’ desired ways of working.

## Introduction

Medications are vital for preventing and treating disease and have contributed to increasing our life expectancy.^1^ At the same time, medications come with risks. Unsafe handling and medication errors are often caused by errors in systems, processes, or in communication about usage.^2–4^ Many of the risks of medication use are preventable.^5–7^

The medication management process requires both communication and information sharing among various professions such as physicians, pharmacists, nurses, and other healthcare professionals, as well as the patient and their relatives.^8,9^ In order to reduce risks related to medications, it is important that everyone involved have access to correct information about a patient's medications.^10^

In Sweden more than 99% of all prescriptions are in electronic form.^11,12^ Regional healthcare in Sweden is decentralized, with self-governing regions and municipal care. In some regions all healthcare providers use the same electronic health records (EHRs), within the region, meaning they have a regionally shared medication list. In other regions there is a large variation in EHRs resulting in a fragmented view of each patients medication.^10,13^ Furthermore the pharmacies use other medication information systems.^14^ Medication lists in the various systems are often not complete and up to date. Reasons for discrepancies between lists can be the fragmented healthcare with different systems, as well as the information that the list is based on and how it is updated, or a lack of responsibility for updating the list.^15,16^ Several Swedish studies have shown that discrepancies among the various sources of information occur often, which can lead to errors and patient safety risks.^11,13,17,18^ A shared national medication list has the potential to increase safety in drug treatment.^11,19–21^ Internationally, several initiatives have aimed at implementing digital shared medication lists.^22,23^

In May 2021, a new law, the National Medication List,^24^*(Lag (2018:1212) om nationell läkemedelslista)* came into force in Sweden. The goal of the National Medication List is to give the patients themselves, healthcare, and pharmacies access to the same information about the patient's prescribed and dispensed medications.^25,26^ The shared medication list is expected to lead to increased patient safety, streamlining of the medication process, decreasing errors in the list and increased patient participation. It is also hoped that the list will make drug abuse more difficult and provide an improved opportunity for structured medication information.^26^

**Figure 1.**
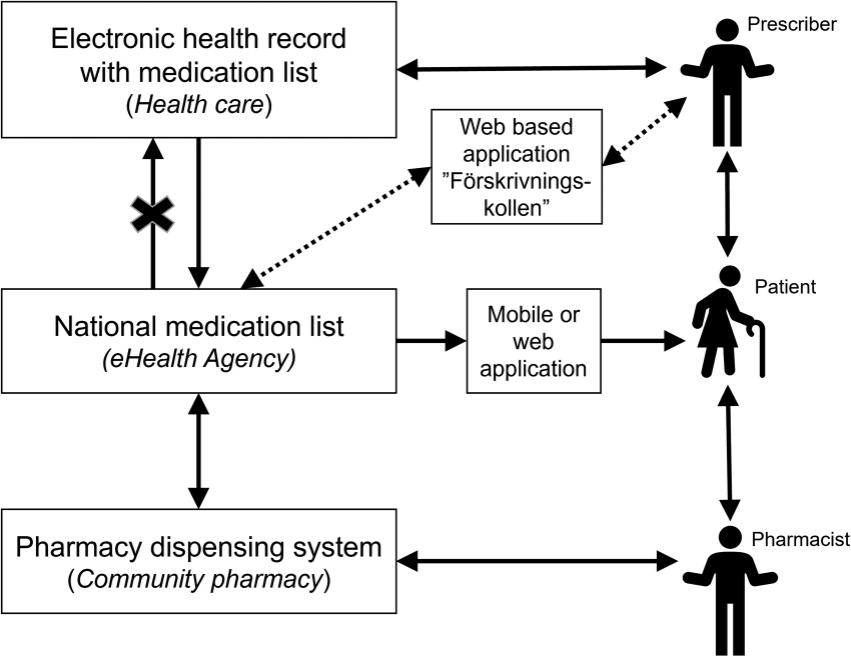
Description of the medication process in Sweden, when patients receive a prescription and get their medications dispensed at a pharmacy. When prescriptions are sent from the EHR, these go directly to the National Medication List. However, the National Medication List is not yet integrated in the EHR, thus healthcare professionals cannot yet see or change the information in the National Medication List in their EHR systems. During this transition period, FK can be used to view the information, prescribe and make changes. However, changes made in FK are not visible in the EHR. EHR: electronic health record; FK: Förskrivningskollen.

The National Medication List is based on prescriptions valid for dispensing medications at pharmacies, not the medication orders (i.e. the decision about treatment documented in healthcare). Furthermore, the list does not include information about medications that are administered at hospitals or nonprescription medications. The National Medication List is being implemented in stages, and it has not yet been integrated into the EHR systems (Figure 1).^26^

In connection with the first stage of implementing the National Medication List, the web-based application Förskrivningskollen (“the Prescription checker”) was launched. Förskrivningskollen (FK) is a service for healthcare professionals that includes information from the National Medication List register, such as a patient's dispensed medication as well as current and historical prescriptions. The information includes dosing instructions, cause of treatment, route of administration and strength, among other things.

FK is intended to function as a backup system until the healthcare system's own EHR systems are integrated with the National Medication List.^27^ In its first version, the user could see information about a patient's prescribed and dispensed medication. In November 2021, functions for prescribing medications were added but is only recommended as a backup or for users who lack another IT system for prescribing medications. Several professions are authorized to use the application, including physicians, nurses, dietitians, midwives, dentists, dental hygienists, and clinical pharmacists.^27^

FK lacks two-way communication with the EHR system, which means that actions carried out in the application are not automatically transferred from the National Medication List to a patient's EHR.^27,28^ To see the information in the application, healthcare professionals need consent from the patient. There are several rules regarding the consent, but in general the consent is verbal and needed each time the user accesses the system. The patient has a right to hide information about specific prescriptions.^29^

Internationally, there are several different initiatives for developing and implementing digital and shared medication lists in order to increase medication safety, although there are variations in terminology, content, access, integration, etc. Implementing a shared national medication list is indeed a complex process with results being affected by technical, human, and organizational aspects.^23,30,31^ New IT systems often pose risks due to difficulties in integrating a new system into existing work processes.^32^ Although there is still limited research investigating the implementation of a shared medication list, available studies show positive effects in the form of increased patient safety as well as risks and challenges during implementation.^22^ A Swedish study carried out just before the National Medication List law came into force highlights the many challenges in the medication management process, and the high expectations among healthcare professionals for (and several concerns about) implementation of the National Medication List.^11,28^

To our knowledge, this is the first study to cover the early phase in the implementation of the National Medication List in Sweden. Studying the users’ perspectives on FK can provide insights that can be used in future implementation of nationally shared medication lists or other IT systems in healthcare.

This study was based on a sociotechnical perspective, where analyses took place based on human/social, organizational, and technical factors, and how these relate to each other.^33^

### Aim of the study

The aim of this study was to examine the users’ experiences and perceptions of FK, to describe benefits, barriers, and possible risks of the application.

More specifically, the study intends to answer the following questions: Who are the users of FK in terms of numbers and professional groups? What knowledge do users have about the National Medication List and FK? What benefits do the users experience? Do users see any barriers to or risks in using FK?

## Method

Data were gathered by a survey with open and closed questions and statistics from the Swedish eHealth Agency about the use of FK.

To improve the validity of the survey questions, cognitive interviews was conducted with the aim to analyze how the respondents understood and interpreted the survey questions.^34^ Six interviews were held with two physicians, three pharmacists, and one nurse. The interviews were held via Zoom, the respondents filled in the survey in real-time and a verbal-probing technique was used to understand their thoughts for each question. After each interview, the questions were adjusted when needed, for example wording, concepts, the order of the questions or the available answers for multiple-choice questions.

The survey was conducted from November 2021 to January 2022 and included both open-ended and closed-ended questions (Table 1). It was distributed with purposive sampling with a focus on reaching physicians, clinical pharmacists, and nurses. In order to identify barriers to using FK, the decision was made to also include healthcare professionals who had not used FK. The study thus includes both the experiences of those who have used the application and the perceptions of those who have not used it.

**Table 1.** Overview of the questionnaire.

Question	Answered by	Type of question	Analysis
1. I work clinically as a…	All respondents	Multiple choice	Quantitative
2. What is your age?	All respondents	Multiple choice	Quantitative
3. What is your gender?	All respondents	Multiple choice	Quantitative
4. Which region do you mainly work in?	All respondents	Multiple choice	Quantitative
5. I mainly work in… [area of work]	All respondents	Multiple choice	Quantitative
6. What is your previous knowledge about the National Medication List?	All respondents	Likert scale 1–5	Quantitative
7. Do you have any expectations or views about the National Medication List?	All respondents	Free-text question	Qualitative
8. How did you find out information about FK?	All respondents	Multiple choice	Quantitative
9. Have you used FK?	All respondents	Yes/no question. Filter question that determines which other questions the respondent answers in the survey	Quantitative
10. What is the reason why you have not used FK?	Those who answered **no** to question 9	Multiple choice	Quantitative
11. Do you have any comments on FK based on what you know today?	Those who answered **no** to question 9	Free-text question	Qualitative
12. How many times have you used FK? (Since the implementation in May 2021)	Those who answered **yes** to question 9	Multiple choice	Quantitative
13. For what purpose have you used FK? (Choose all that apply)	Those who answered **yes** to question 9	Multiple choice	Quantitative
14. FK has added value to my clinical work.	Those who answered **yes** to question 9	Degree of agreement 1–5 Describe or give examples in free text	Quantitative Qualitative
15. Is there anything you find difficult or unclear in the use of FK?	Those who answered **yes** to question 9	Yes/no/don’t know Describe or give examples in free text	Quantitative Qualitative
16. Do you see any risks regarding the use of FK?	Those who answered **yes** to question 9	Free-text question	Qualitative
17. Is there anything else you would like to add?	All the respondents	Free-text question	Qualitative

The questions were written in Swedish. There was a free-text comment field in connection with all questions, except for questions 2 and 3. For all questions with fixed answer options, there was an option of choosing “other” or “don't know.” FK: Förskrivningskollen.

The survey was distributed via professional associations in areas of medicine where handling of medication information is especially important. The survey was also distributed via medically responsible nurses within municipal care and via medication committees in the regions of Sweden. The survey was mediated directly via e-mail, via publication in newsletters or via social media. It was also published on the FK website November 3, 2021 through January 1, 2022.

The study was based on a convergent mixed methods design, with quantitative and qualitative data analyzed separately and then interpreted and integrated as a whole.^35^

All analyses of the survey's quantitative data were carried out in IBM SPSS Statistics 27 and Microsoft Excel 365. To analyze whether the difference between groups was statistically significant, a Mann-Whitney U-test was used for two of the questions, with a significance level of p < 0.05. The null hypothesis was defined as no difference between the groups. The qualitative data analyses were based on an inductive approach. The free-text responses were analyzed with a qualitative content analysis.^36,37^ The analysis was made by the first author (FB), however all categories were discussed and critically reviewed by both the authors.

The study was approved by the Swedish Ethical Review Authority as a part of a larger project about effects from the implementation of the National Medication List (Dnr 2019-06553, decision 2020-03-09). The respondents received written information about the purpose of the study, their anonymity, and security in data handling. Respondents were assured that it would not be possible to identify any individuals or discern any details about them in presentations of the results.

## Results

### Statistics on use of the application

**Figure 2.**
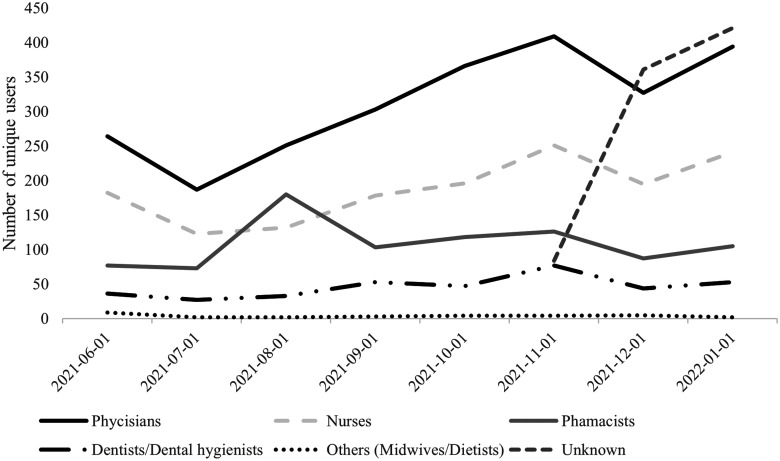
Number of unique users of FK during the period May 2021 to January 2022 by profession. The group “unknown” had logged in outside healthcare facilities, which is interpreted as physicians prescribing outside an employment. FK: Förskrivningskollen.

Statistics from the Swedish eHealth Agency showed that FK was used by an average of 755 unique users per month during the period May 2021 (when the service was first launched) through January 2022. To put the number of unique users into perspective, there were a total of 41,485 physicians and 111,647 nurses working in the area of healthcare in Sweden in 2020.^38^ Users came from all of Sweden's 21 regions and 7 different professions (Figure 2). The application was also used by retired physicians or physicians who were prescribing medication during off hours or outside an employment [In Swedish: fritidsförskrivare].

### Description of respondents

The study's questionnaire was answered by a total of 288 respondents. The most common profession was physicians, followed by nurses and clinical pharmacists (Table 2). Of 288 respondents, 84 respondents had used FK. Of the 84 who used FK, 57% were clinical pharmacists, 37% physicians, and 6% other professions.

**Table 2.** Description of the survey respondents with distribution based on gender, age, profession, and field of work (n  =  288).

Background factor	Alternative	Quantity	Percentage
Gender	Man	91	31.6
	Woman	188	65.3
	Other/do not want to specify	4	1.4
	No answer	5	1.7
Age	Younger than 30 years	15	5.2
	30–39 years	69	24.0
	40–49 years	77	26.7
	50–59 years	74	25.7
	60 years or older	50	17.4
	Do not want to specify	1	0.3
	No answer	2	0.7
Profession	Clinical pharmacist	65	22.6
	Physician	123	42.7
	Nurse	83	28.8
	Dentist	8	2.8
	Physician prescribing outside healthcare	3	1.0
	Other	3	1.0
	Not answered	3	1.0
Work area	Hospital-based care	106	36.8
	Primary care	66	22.9
	Specialist care	44	15.3
	Municipal care	60	20.8
	Other	12	4.2

A majority (74%) of the clinical pharmacists included in the study worked in hospital-based care, while a majority (66%) of the nurses included in the study worked in municipal care. Physicians were more evenly distributed in different areas, where hospital-based care was the most common. All 21 regions in Sweden were represented among the respondents in the study.

### Quantitative results from the surveys

**Figure 3.**
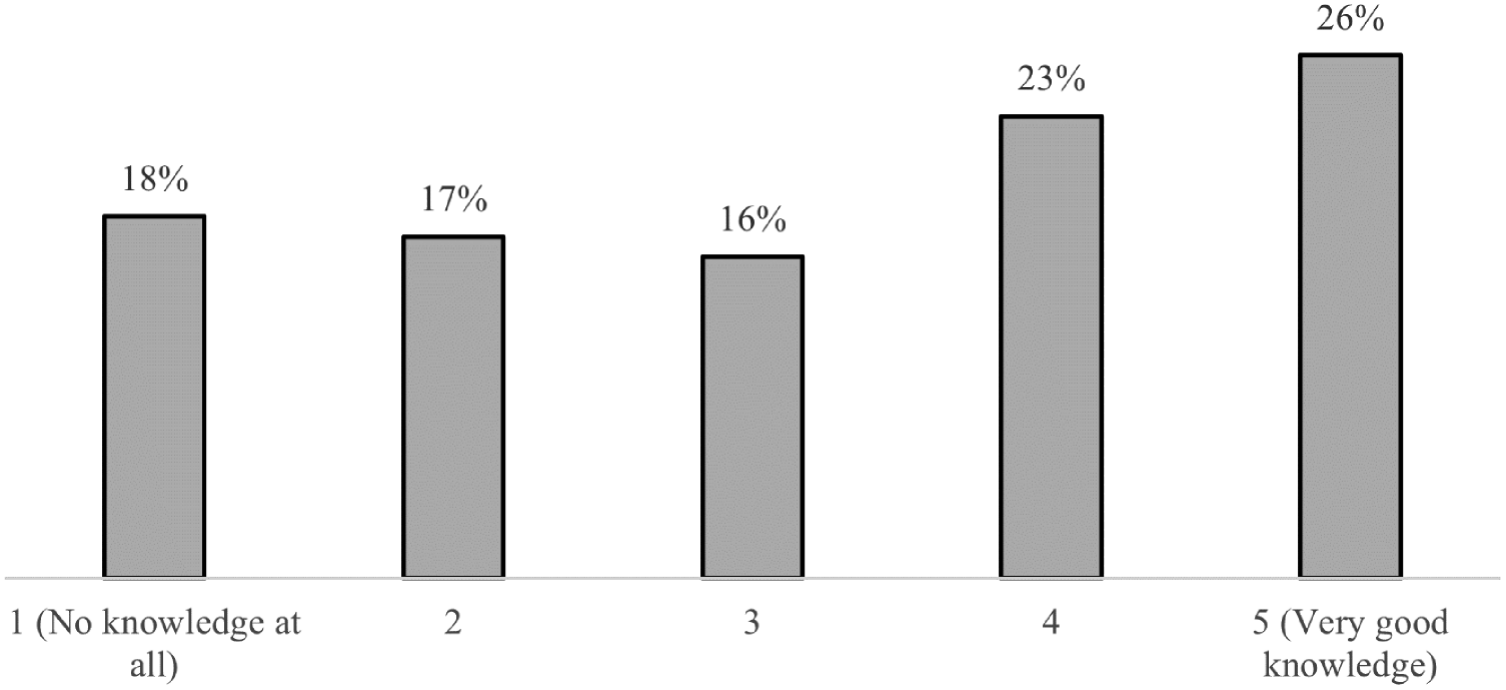
Response distribution (%) based on the question “What is your previous knowledge about the National Medication List?”. The respondents estimated their knowledge from 1 to 5, where 1 stood for “no knowledge at all” and 5 for “very good knowledge.”

One of the questions was about how much the respondents knew about the National Medication List (Figure 3). The results indicate a large diversity in answers on the five-point Likert scale, with answers ranging from 1, representing “no knowledge at all” to 5, representing “very good knowledge.” The majority of the clinical pharmacists included in the study (97%) estimated high knowledge (4 or 5), while there was a greater diversity of responses from the physicians and nurses. The difference between the groups was statistically significant (p < 0.05, Mann-Whitney U-test). Among those working in municipal care, 68% had estimated a low knowledge (1 or 2) of the National Medication List, while 66% of those working in hospital-based care estimated a high knowledge (4 or 5). The difference between the groups was statistically significant (p < 0.05, Mann-Whitney U-test) among all work areas, except between primary care and specialist care.

One of the questions covered how the respondents had received information about FK, to which 36% responded with the option “have not received any information about FK,” 23% responded “via employer,” and 28% responded “via colleagues.”

**Figure 4.**
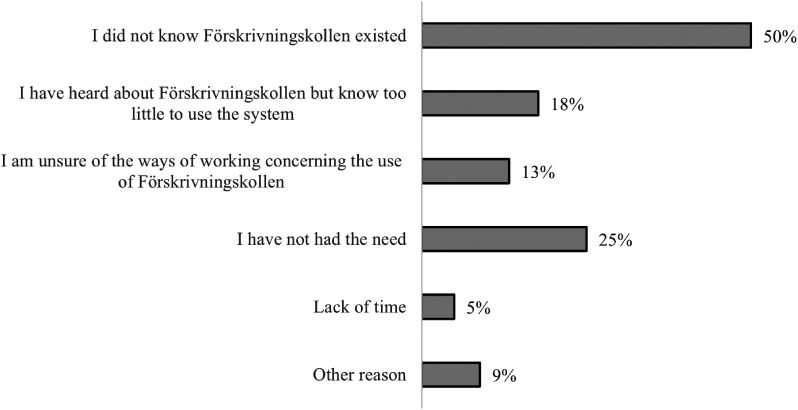
Response distribution (%) based on the question “What is the reason why you have not used FK?” It was possible to choose several options. Only respondents who stated that they have not used FK answered this question (n  =  204). FK: Förskrivningskollen.

Of the respondents who had never used FK, half said that the reason was that they did not know that FK existed (Figure 4).

Of the respondents who had used FK (n  =  84), the most common purposes for using FK were connected to medication reconciliation or medication review (67%), while 31% said that they used it as a source of information when prescribing medications.

Respondents who had used FK reported a lot of variation in how often they had used the application since it was implemented. Most respondents said they had used it 1–2 times (31%) or 3–10 times (32%), but 25% had used it 11–50 times, and 11% more than 50 times.

**Figure 5.**
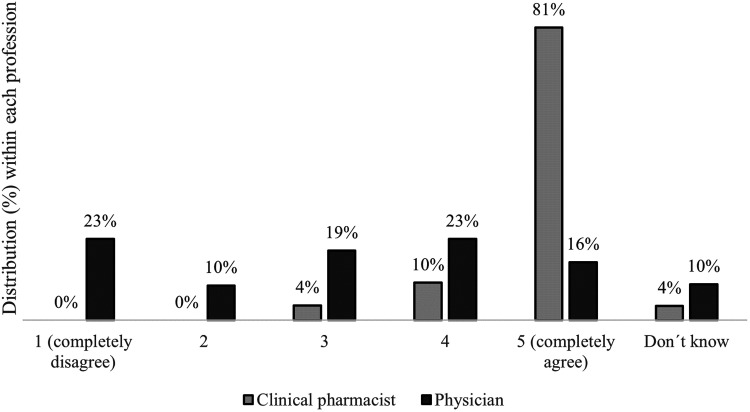
Respondents’ degrees of agreement with the statement “FK has benefitted my clinical work” according to profession. Agreement was indicated on a scale of 1 to 5 (1  =  completely disagree; 5  =  completely agree). Response distribution (%) within each profession. Other professional categories are not shown due to the low number of respondents (n  =  6). FK: Förskrivningskollen.

Most respondents who had used FK (54%) said that they fully agreed with the statement “FK has benefitted my clinical work.” A few (8%) indicated that they did not agree at all with the statement regarding benefit. A majority (81%) of the clinical pharmacists stated that they fully agreed with the statement, while physicians had a greater spread in their responses (Figure 5). The difference between physicians and clinical pharmacists regarding estimated benefit was statistically significant (p < 0.05, Mann-Whitney U-test).

### Qualitative results from the surveys

Qualitative analysis of the open-ended (free text) answers and comments on the other questions resulted in six main categories and 18 subcategories (Table 3).

**Table 3.** Main categories and subcategories that emerged from analysis of the open-ended answers and comment fields.

Main category	Subcategory
Expectations—National Medication List	Safer medication treatment due to correct information A shared picture among all people involved Simple and easy to use Well-functioning procedures for updating the list
Concerns—National Medication List	Includes only prescriptions dispensed at pharmacies Concerns about parallel systems Systems are developed without sufficient knowledge of healthcare needs Concerns about patient consent and a patient's right to hide information
Benefit of FK	Simplifies the work of clinical pharmacists Information on prescriptions from other healthcare providers
Lacks knowledge about FK	Do not know about FK Uncertainty about routines, use, and regulations concerning FK
Barriers to using FK	Unclarity in user interface A nonupdated list Time-consuming Prefer to use other systems
Potential risks of FK	Lack of clinical documentation False sense of security

FK: Förskrivningskollen.

The respondents described the problems that currently exist in the medication process in Sweden, such as incorrect lists and lack of information.

“Hopefully it will become clearer which medication the patient uses and is prescribed. Currently, there is no proper way to see which medications a patient is prescribed and is taking if the patient themself cannot account for this.”—Nurse

The respondents had expectations of a safer medication treatment with a reduced risk of incorrect medication, double dosing, interactions, and safer prescription of addictive drugs.

“We sometimes have patients who seek both dental care and primary care to get narcotic painkillers, aware that we do not have access to other electronic health records.”—Dentist

The respondents expressed a concern that the list would not be complete since medications administered at hospital and nonprescriptions medications are not included.

“The most negative thing is that it is called a National Medication List, which can lead patients to believe that it is actually a list of current medications and not only prescriptions dispensed at the pharmacy.”—Physician

There were concerns about parallel systems, that is, that the National Medication List would become a system on the side and that it would simply double the required effort for the healthcare professionals. Respondents also expressed concerns about the regulations connected to the National Medication List (i.e. the need for patient consent and the possibility for patients to hide information about specific medications) and their effects on patient safety.

“For me as a prescriber, it is important to be able to see what the patient has been prescribed and what has been dispensed, in order to be able to take responsibility for the safety of the medication treatment. However, I am uncertain about which has higher priority, that or the patient's right to withhold the same information from the prescriber…”—Physician

Several clinical pharmacists described great benefits perceived by using FK. The application had facilitated their work, as they previously had to turn to a physician or nurse to access information about dispensed prescriptions.

“Use pretty much daily. An extremely valuable tool for pharmacists when working with medication reconciliations. Finally, we have access to information about dispensed medications!”—Clinical pharmacist

Another benefit highlighted by the physicians was access to information about prescriptions from other healthcare providers, especially in connection with the prescription of addictive drugs. Other physicians said that they preferred to use other national systems, such as Nationell patientöversikt (NPÖ; “National Patient Overview”) or Pascal. In NPÖ, healthcare staff can access information from other EHRs, though NPÖ does not include information from all healthcare providers or all information from the EHR. Pascal is a web-based system used for prescribing and handling patients with multidose drug dispensing. In Pascal, it is also possible, with the patient's consent, to see information about dispensed medication for patients with ordinary prescriptions (nonmultidose).

Several respondents said that they had not heard about FK. Other respondents described an uncertainty regarding work routines and regulations in connection with the use of the application.

“Despite really trying to understand, I don't understand routines and it feels very unsafe as prescriptions made in the application are not visible in the patient's electronic health record.”—Physician

Some of the respondents noted that the list was not updated, and that there were duplicates and outdated prescriptions in the list, which made it difficult to use. Several physicians described FK as time-consuming, primarily based on the need for separate logins and patient consent.

Some respondents were concerned that prescriptions or changes made in FK would not be documented in the patient's EHR. Another concern was that FK could lead to a sense of false security if healthcare professionals rely on the list without checking with the patient.

“Yes, the list is not updated. Many out-of-date prescriptions or out-of-date doses can be found in The National Medication List… [Because of that] Incorrect doses and drugs have been added to the medication list for hospitalized patients several times in the department as one can get the impression in the National Medication List that the prescriptions are valid.”—Clinical pharmacist

## Discussion

Our results highlights several challenges in the implementation of the National Medication List, from a technical, human, and organizational perspective, which is in line with previous research on shared medication lists.^22,28,31,39^ Many of the perceived barriers that emerged were related to the working processes that would be required to reach the expected benefits. These concerns included regulations about patient consent, the patients’ rights to hide specific medications, who has the responsibility for updating the list, and how clinical documentation should take place. The results show that FK has users in several different professions and in different work areas within healthcare. The National Medication List has even more kinds of users, like patients, relatives, and pharmacy staff. Thus, the medication process involves users with different backgrounds, roles, and needs.

The respondents’ expectations and concerns are similar to what healthcare professionals described in an earlier study before implementation of the National Medication List.^28^ The respondents expect well-functioning routines for updating the National Medication List.

Studies have shown that there are different opinions regarding who has the responsibility for a patient's medication list.^10,16^ Several studies highlight the importance of clarifying routines and responsibilities regarding a patient's prescribed medications if a shared medication list is to be implemented.^10,21,31^

Other concerns raised by the respondents are about the new regulations regarding patient consent and right of a patient to hide information about specific medications. The result highlight that respondents are uncertain of how regulations should be interpreted and applied in connection with the use of FK. This issue has been raised previously in a debate article discussing that the regulations may be difficult for both patients and healthcare staff to understand.^40^ A major challenge is that there are two different laws with different principles that regulate how the patient can hide information. These raised concerns are also in line with a study that shows that consent and rules around it can be interpreted in different ways, and that healthcare professionals are concerned about the ethical aspects surrounding this issue.^39^ In Sweden there is a need to clarify and agree on a common process for working with medications and taking responsibility for patients’ medication list, which the Swedish Society of Medicine are currently working on.

Another concern that has also previously been highlighted by healthcare professionals is that the National Medication List does not include all of a patient's medications.^28,40,41^ Sweden's National Medication List is based on prescriptions (for dispensing at pharmacies) and not on medication orders corresponding to decisions made for the patient's entire medication plan (which is documented and maintained in the EHR). Some decisions about medication treatment, like changing of dose, medication, or termination of treatment, is not communicated with a new prescription and will therefore not be updated correctly in a list based on prescriptions. Thus, the National Medication List cannot be expected to be correct and up to date before it is fully integrated with healthcare EHRs. Furthermore, some medications are provided by healthcare without any prescription and will not be included even when it is fully integrated. In addition, the responsibilities for making sure the information about prescriptions in the shared register is correct are not clear, the National Medication List is not intended to be used as a medication list for patients. Instead, patients should still use a medication list (based on medication orders) printed from the EHR.

Our results show that FK has few users among all healthcare staff who have access to the information in the application. Our results suggest that one reason could be that healthcare staff simply do not know about the application. FK was initially intended only to be a backup system, and the ambition may never have been widespread use. At the same time, FK is used in daily patient work, which means there is a need to clarify working routines linked to the application. The need for information and clarification of working routines was also highlighted in the risk analysis of the National Medication List carried out by the network Sweden's Chief Physicians.^42^

Several problems raised by the respondents in this study are a consequence of the lack of interoperability between FK and EHR. The respondents said that FK is time-consuming because it requires that several steps be performed repeatedly. That healthcare professionals highlight integration with medical records as decisive for how well a common medication list will work is in line with previous research.^28,31^

A majority of the clinical pharmacists who participated in this study described the benefit using FK, while the physicians who used the application were more divided about perceived benefit. These two groups of healthcare professionals have very different roles in the treatment of the patients, and therefore use the medication list in different ways. Part of the difference between clinical pharmacists and physicians seems to be explained by the fact that the clinical pharmacists have previously not been authorized to use the same system as prescribers to view dispensed prescriptions. Previous research examining the perspective of healthcare professionals in Austria when implementing a national drug list demonstrated differences among healthcare professions in use, acceptance and needs.^43^

Even when the National Medication List is integrated and hopefully provides a correct list of current medications it is important to remember that it will not provide a complete picture of a patients’ medication treatment and information relevant in making decisions related to it. Prescribers sometimes also require other more descriptive information from the EHR, to understand reasoning behind treatment decisions and to follow adjustments and changes in the medication's treatments.^10^ To get the complete picture it would also be valuable to increase patient involvement and open for possibilities for patients to add their over-the-counter (OTC) medications. Furthermore, even if a shared medication list for all healthcare providers can lead to a more consistent picture of a patient's prescriptions, there will still be a need for medication reconciliation with the patient or relatives.^21^

### Strengths and weaknesses of the study

By using a survey, the study captured a diversity of perspectives and was able to include respondents from different work areas, professions, and geographic locations. The mixed method contributed to a deeper understanding of the healthcare professionals’ experiences and perceptions. There are several examples of quantitative results beings both explained and strengthened by free-text answers. For example, the quantitative results demonstrated differences among groups of respondents, while the free-text answers created an in-depth picture of what types of benefit the respondents saw. The open-ended questions provided the opportunity to see patterns and themes that were not possible to predict when the survey was constructed.

The purposive sampling meant that the survey could be administered in areas where medication issues are particularly important. This sampling may have contributed to the fact that the survey reached some workers who actually use FK, and perhaps these respondents were motivated to express their opinions. At the same time, the purposive sampling produced a skewed distribution within different subgroups of respondents. For example, 66% of the nurses sampled here work in municipal care, while statistics (from 2020) show that the actual percentage of nurses in municipal care is about 20%.^44^ Also it is likely that a selection bias affected the results by perhaps including respondents with more knowledge about the topic than the average, thus limiting the generalizability.

The survey was constructed in several stages with the aim of strengthening the validity and reliability of the survey questions. Cognitive interviews were important for investigating how the questions would be interpreted and understood. The interviews provided an opportunity to adapt the questions based on the perspectives of the various professionals.

Both the quantitative and qualitative analyses were conducted systematically. In the qualitative analysis, the categories were continuously checked against the previous steps in the process and the categories were discussed to achieve consensus.

### Future research

Based on the results of this study, there is a need for continued research to follow up barriers and risks in future phases of the implementation of the National Medication List. The respondents’ concerns highlight several ways in which the National Medication List affects patient safety. Future research should evaluate how well the expectations for increased patient safety in medication treatment are met. There is also need for research following up risks, such as a potential lack of clinical documentation, safety issues regarding regulations adjacent to the National Medication list, and whether the National Medication List can lead to a false sense of security. In addition, future studies should compare Sweden's efforts in this area to initiatives from other countries.

## Conclusion

Our results showed that expectations are high for the National Medication List in Sweden, but that there are several concerns about implementation. At the time of the survey, there was a low level of knowledge about the web-based application FK and uncertainty regarding working routines and the regulations connected to the application.

The lack of interoperability with the EHR systems made FK time-consuming and posed a risk that clinical documentation would be lacking. Respondents said that the medication list in FK was not updated and there was a concern that FK could lead to users having a false sense of security about the accuracy of the list.

The majority of the clinical pharmacists sampled here thought that FK added benefit because it facilitated their work with medication reconciliation. The physician group was more ambivalent about the benefits of FK, with some indicating that FK was useful when prescribing addictive drugs, while others did not see much benefit and preferred to use other systems.

Overall, there is a need for information about, and clarification of, working routines and regulations linked to FK and the National Medication List. The concerns professionals have given provide important insights into how shared medication lists should be implemented. In Sweden, the potential value of the national, shared medication list will probably not be realized until it is fully integrated into the EHR in a way that supports healthcare professionals’ desired ways of working.
